# A Systematic Review and Meta-Analysis of Bag Extraction Versus Direct Extraction for Retrieval of Gallbladder After Laparoscopic Cholecystectomy

**DOI:** 10.7759/cureus.35493

**Published:** 2023-02-26

**Authors:** Hussam Khougali Mohamed, Mohamed Albendary, Ali Ahmed Wuheb, Omer Ali, Mohammed Jibreel Mohammed, Mohamed Osman, Mohamed S M Elshikhawoda, Ali Yasen Mohamedahmed

**Affiliations:** 1 General and Upper Gastrointestinal Surgery, University Hospital Hairmyres, Glasgow, GBR; 2 General Surgery, Peterborough City Hospital, Peterborough, GBR; 3 Surgery, The Royal Wolverhampton NHS Trust, Wolverhampton, GBR; 4 Surgical Oncology, Blackburn Royal, Lancashire, GBR; 5 General and Colorectal Surgery, University Hospital Wishaw, Glasgow, GBR; 6 General Surgery, Princess of Wales Hospital, Bridgend, GBR; 7 Vascular Surgery, Glan Clwyd Hospital, Rhyl, GBR; 8 General Surgery, The Royal Wolverhampton NHS Trust, Wolverhampton, GBR

**Keywords:** specimen bag, bile spillage, port site infection, laporoscopic cholecystectomy, gallbladder removal

## Abstract

This analysis aims to evaluate the comparative outcomes of gallbladder extraction with a bag versus direct extraction in laparoscopic cholecystectomy (LC). A systematic online search was conducted using the following databases: PubMed, Scopus, Cochrane database, The Virtual Health Library, Clinical trials.gov, and Science Direct. Comparative studies comparing bag versus direct extraction of the gallbladder in LC were included. Outcomes were surgical site infection (SSI), the extension of fascial defect to extract the gallbladder, intra-abdominal collection, bile spillage, and port-site hernia. Revman 5.4 (Cochrane, London, United Kingdom) was used for the data analysis. The results show eight studies were eligible to be included in this review with a total number of 1805 patients divided between endo-bag (n=835) and direct extraction (n=970). Four of the included studies were randomized controlled trials (RCTs) while the rest were observational studies. The rate of SSI and bile spillage were significantly higher in the direct extraction group: odds ratio (OR)=2.50, p=0.006 and OR=2.83, p=0.01, respectively. Comparable results were observed regarding intra-abdominal collection between the two groups(OR=0.01, p=0.51). However, the extension of a fascial defect was higher in the endo-bag group (OR=0.22, p=0.00001), and no difference was observed regarding the port-site hernia rate (OR-0.70, p=0.55). In conclusion, extraction of the gallbladder with an endo-bag provides a lower rate of SSI and bile spillage with similar results regarding post-operative intra-abdominal collection. Although with the endo-bag, the fascial defect will more likely need to be increased to extract the gallbladder. However, the port-site hernia rate remains similar between the two groups.

## Introduction and background

Since the introduction of laparoscopic cholecystectomy (LC) in the 1980s, it has become the gold standard treatment for gallbladder disease [1]. Laparoscopic cholecystectomy has proven tremendous outcomes in minimizing the rates of postoperative morbidity, hospital stay, and fast recovery [2,3]. Perforation of the gallbladder during surgery is considered a leading cause of bile and stone spillage, which is still not uncommon in LC either during dissection or specimen extraction resulting in an increased risk of surgical site infection (SSI) [4,5]. While port site-related morbidity following LC remains of a low incidence compared to wound problems in open surgery, it is still reported as a frustrating complication that undermines the benefits of laparoscopic surgery [3,5]. Moreover, the financial burden of managing such complications is inevitable.

Port site complications were reported to be 10.5% and varied from bleeding (4%), infection (2.5%), and hernia (1.5%) [6]. Hence, alternative techniques were implemented to lessen port site morbidity, particularly wound infection, such as adopting the closed approach of pneumoperitoneum and using different retrieval devices [6,7]. Although retrieval endo-bags may facilitate the safe extraction of the gallbladder, the cost and risk of widening the port site and subsequent hazards of abdominal wall hernias are ongoing concerns [1,2,5]. With no significant benefits of the use of endo-bags in cholecystectomy, apart from cases of cholecystitis and cancer, the economic cost may influence the standard use of retrieval bags, especially in developing countries and their struggling healthcare systems [4,7]. 

Therefore, this analysis was designed to evaluate the routine use of retrieval bags in LC, the potential risks and benefits as well as to determine whether the routine use of endo-bags for gallbladder extraction is mandatory or not.

## Review

Methods

This systematic review and meta-analysis were done prior to the inception of the study using the Preferred Reporting Items for Systematic reviews and Meta-Analyses (PRISMA) guidelines [8,9].

Inclusion and Exclusions Criteria

Table 1 features the inclusion and exclusion criteria applied to this systematic review and meta-analysis.

**Table 1:** Inclusion & exclusion criteria LC: Laparoscopic cholecystectomy

Inclusion criteria	Exclusion criteria
Randomized controlled trials or comparative observational studies	Non-comparative studies, case series, case reports, and letters
Including patients of all age groups of any gender	Open cholecystectomy where no comparison is made between bag and no bag extraction
Including patients with LC	
Comparing the outcomes of the bag group with the direct extraction group for LCs	

Search Strategy

A literature search was systematically undertaken from Medline, Embase, PubMed, Research Gate, and Google Scholar by two authors independently. The electronic searches were performed using a combination of the following search terms: ‘gallbladder’, ‘laparoscopic cholecystectomy’, ‘specimen’, ‘retrieval, ‘extraction’, ‘endo-bag’, ‘direct extraction’. Studies enrolling patients of any age or gender were included from inception until July 2022. The references of relevant reviews and included studies were also checked manually to identify additional potentially eligible studies. The searches were limited to human subjects and no language restrictions were applied during the search of all electronic databases.

Data Collection and Outcomes

Data from included studies were extracted by one author using a standardized data collection sheet. Two other independent authors were asked to check and review the extracted data and a fourth author was asked to resolve any disagreements.

The primary outcome was SSI. Extension of the fascial defect in order to extract the gallbladder, intra-operative bile spillage, post-operative intra-abdominal collection, and port site hernia were considered secondary outcomes.

Assessment for Risk of Bias

The Cochrane risk of bias tool was used to appraise the risk of bias for the randomized trials [10]. Two investigators independently reviewed all studies and graded the risk as 'high', 'low' or 'unclear' in the following categories: random sequence generation, allocation concealment, blinding of participants and personnel, blinding of outcome assessment, incomplete outcome data, selective reporting, and other sources of bias.

The Newcastle-Ottawa scale (NOS) was used to assess the risk of bias in observational studies [11]. Studies were considered low, medium, or high risk of bias if the NOS was 9, 7 or 8, or <6, respectively. Disagreement was resolved with discussion and if they remained unresolved, a third investigator was consulted.

Statistical Analysis and Data Synthesis

This meta-analysis was performed using RevMan version 5.4 (Cochrane, London, United Kingdom). Dichotomous outcomes were pooled with a random effects mode and the odds ratio (OR) with 95% confidence intervals (CI) was estimated. The risk ratio (RR) or risk difference (RD) is the risk of an adverse event in the direct extraction group compared to the bag extraction group.

The heterogeneity was assessed using Cochrane's Q, and quantified using the I2 score. Furthermore, an I2 score greater than 50% indicates significant data heterogeneity. Results were considered statistically significant if p<0.05.

A sensitivity analysis was performed by estimating the RR and RD for each defined outcome. Add to that, we evaluated the effect of each study on the overall effect size and heterogeneity by repeating the analysis excluding one study at a time [12].

Results

**Figure 1:**
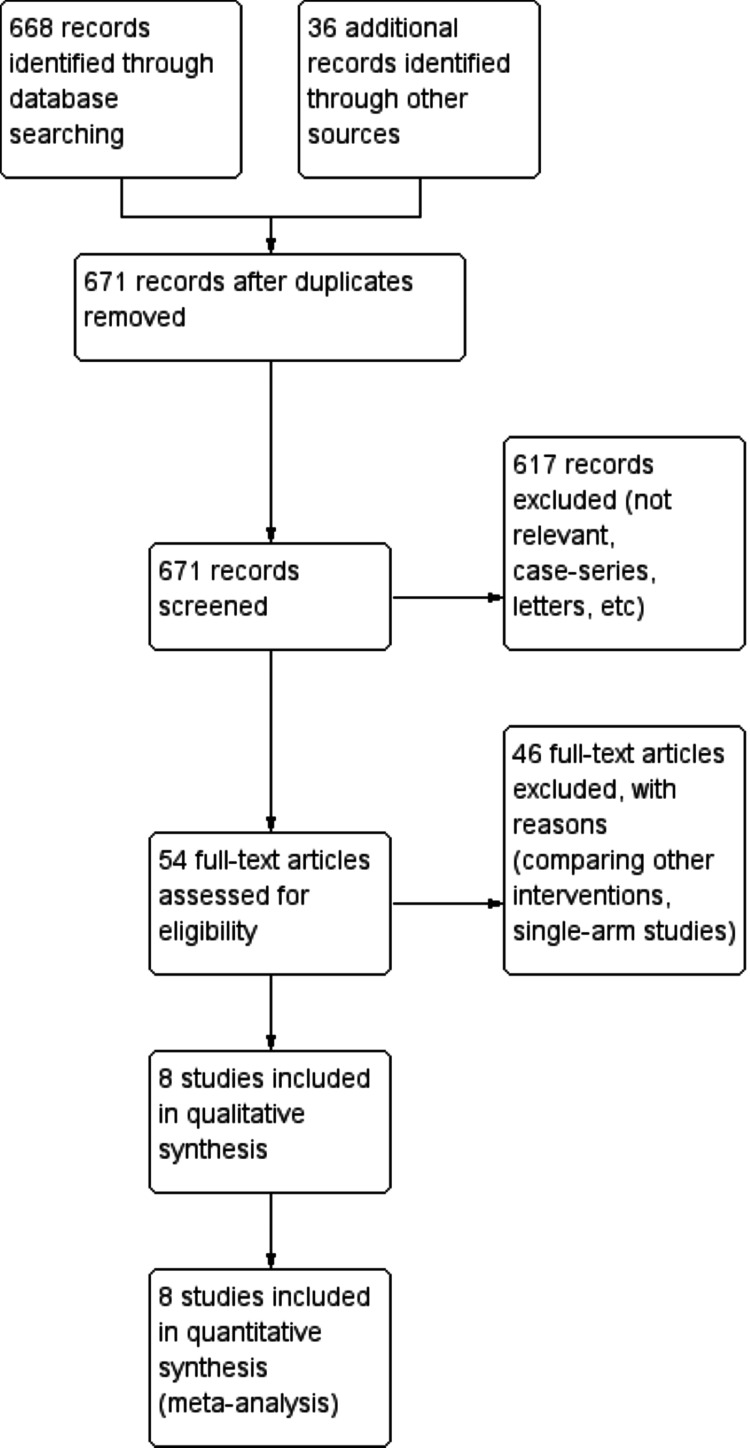
The PRISMA flow chart PRISMA: Preferred Reporting Items for Systematic Reviews and Meta-Analyses

A total of 671 studies were identified after the systemic search of the above-mentioned databases. Of these 54 studies, eight studies were eligible to be included in this systematic review and meta-analysis [13-20]. The PRISMA flow chart is shown in Figure 1. The total number of patients in the eight included studies was 1805 patients. Those patients either had their gallbladders extracted through a bag method (n=831) or extracted directly (n=974). Four of the included studies were randomized controlled trials (RCTs) [14,15,17,20], while the rest were observational studies [13,16,18,19]. A sterile surgical glove was used to extract the gallbladder specimen in two studies [15,17] whereas different types of sterile endo-bags were used as the methods of extraction in the remaining studies.

**Figure 2:**
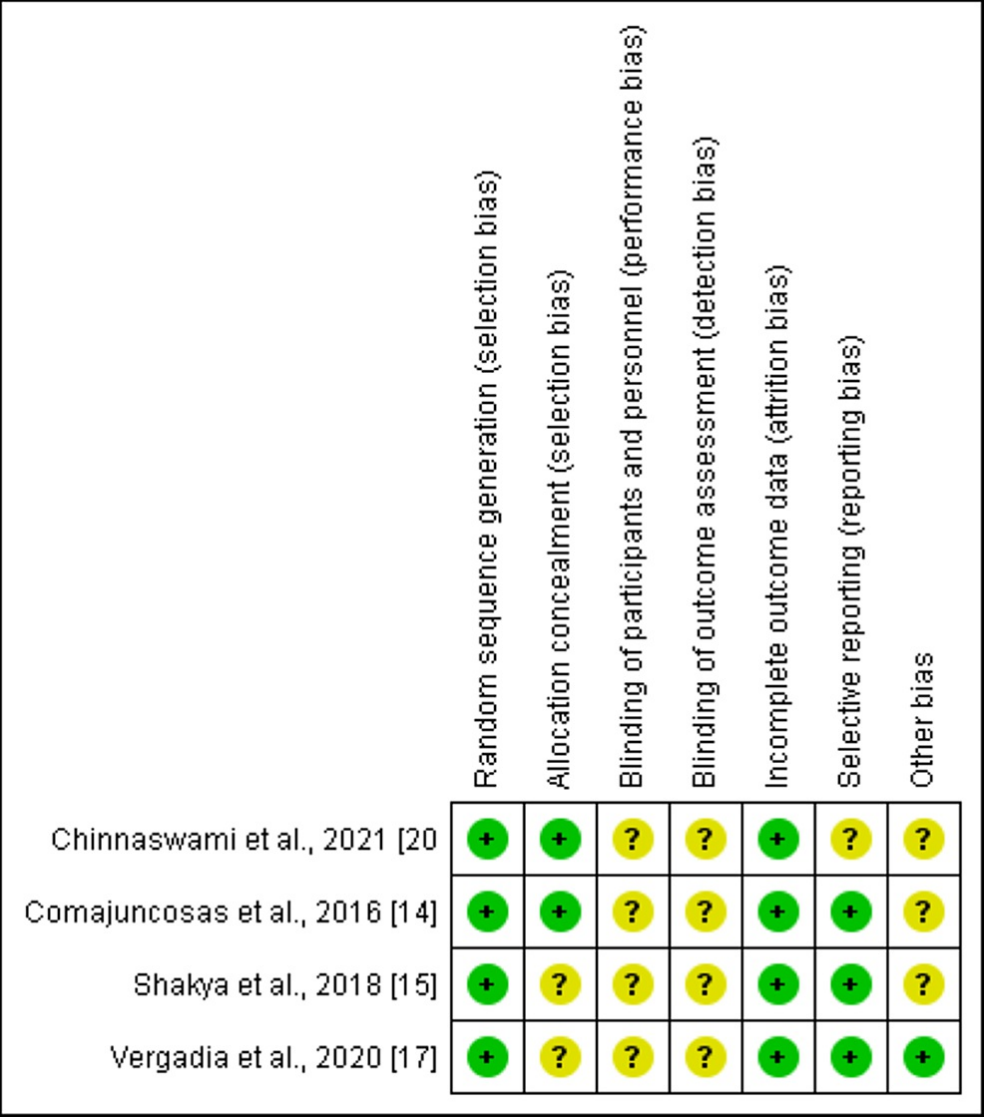
Risk of bias summary for included RCTs RCTs: Randomized control trials

Regarding prophylactic antibiotics, one dosage of antibiotics was given in one study [13], and three doses were reported in another different study [20]. In all the included studies, the specimen was extracted through the epigastric port except in one study in which the umbilical port was used [13]. Characteristics of the included studies are shown in Table 2. Risk of bias assessment is summarized in Figure 2 for the RCTs, and in Table 3 for the observational studies.

**Table 2:** Characteristics of the included studies LC: Laparoscopic cholecystectomy, CBD: Common bile duct

Study	Country	Type of the study	Number of patients	Method of extraction in the bag group	Inclusion and exclusion criteria
Majid et al., 2016 [13]	Ireland	Prospective cohort study	Total: 373, Bag group: 152, Direct extraction group: 221	Endo-bag	Inclusion criteria: Patients undergoing planned elective day case LC. Exclusion criteria: Bile or stone spillage during the procedure were excluded as this necessitated the use of a retrieval bag.
Comajuncosas et al., 2016 [14]	Spain	Randomized clinical trial	Total: 156, Bag group: 76, Direct extraction group: 80	Endo-bag	Inclusion criteria: Patients older than 18 years with uncomplicated or low-risk cholelithiasis who underwent elective LC. Exclusion criteria: Patients who did not consent to the study; had bile duct stones, previous surgery or obstruction of CBD, or recent cholecystitis; used antibiotics seven days before or after the operation.
Shakya et al., 2018 [15]	India	Randomized control trial	Total: 100, Bag group: 50, Direct extraction group: 50	Sterile rubber glove	Inclusion criteria: All the patients with proven cholelithiasis on ultrasound scan and fit for surgery. Exclusion criteria: Associated liver/renal pathology, carcinoma or obstruction of the gallbladder, and any functional or psychiatric disorder.
Singh et al., 2018 [16]	India	Prospective cohort study	Total: 100, Bag group: 50, Direct extraction group: 50	Sterile plastic bag	Inclusion criteria: All patients diagnosed to be having cholelithiasis. Exclusion criteria: Patients having a mass felt on examination.
Vergadia et al., 2020 [17]	India	Randomized control trial	Total: 50, Bag group: 25, Direct extraction group: 50	Sterile surgical glove	Inclusion criteria: All patients with symptomatic cholelithiasis willing for cholecystectomy with the laparoscopic approach. Exclusion criteria: Gallbladder gangrene, pyocele perforation, immunocompromised patients such as those with AIDS, cancer, and usage of steroids.
Sherwani et al., 2020 [18]	India	Comparative prospective study	Total: 600, Bag group: 288, Direct extraction group: 312	Polythene-covered plastic bag	Inclusion criteria: All patients underwent elective LC.
Qassem et al., 2021 [19]	Egypt	Prospective cohort study	Total: 100, Bag group: 50, Direct extraction group: 50	Inzii retrieval system	Inclusion criteria: Age <65 with acute cholecystitis. Exclusion criteria: Concomitant CBD stones, severe uncontrolled comorbidities with American Society of Anaesthesiology score ≥ III.
Chinnaswami et al., 2021 [20]	India	Randomized control trial	Total: 326, Bag group: 140, Direct extraction group: 186	Sterilized plastic bag	Inclusion criteria: All adult patients above 18 years. Exclusion criteria: Empyema of the gallbladder and converted to open cholecystectomy.

**Table 3:** Risk of bias assessment with the Newcastle-Ottawa scale Symbol (*) is the number of points given to each study according to the Newcastle-Ottawa scale (* gives one point, and ** gives two points).

Study	Representativeness of the exposed cohort	Selection of the non-exposed cohort	Ascertainment of exposure	Demonstration that outcome of interest was not present at the start of the study	Comparability of cohorts based on the design or analysis controlled for confounders	Assessment of outcome	Was follow-up long enough for outcomes to occur?	Adequacy of follow-up of cohorts	Total
Qassem et al., 2021 [18]	*	*	*	*	*	*	*	*	8
Sherwani et al., 2020 [17]	*	*	*	*	**	*		*	8
Singh et al., 2018 [20]	*	*	*	*	**	*	*	*	9
Majid et al., 2016 [15]	*	*	*	*		*	*	*	7

Surgical Site Infection

**Figure 3:**
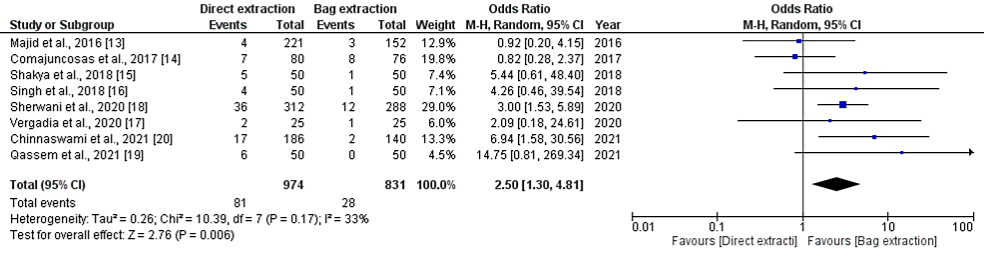
Forest plot analysis of SSI SSI: Surgical site infection, CI: Confidence interval

Port-site SSI was reported in eight studies with a total number of 1805 patients. Surgical site infection was observed in 109 patients (6%) in total (Figure 3). The direct extraction group showed a statistically higher rate of SSI compared to the bag group (8.3% versus 3.5%, OR 2.50, 95% CI (1.30, 4.81), p=0.006). The level of heterogeneity was low among the included studies (I2=33%, p=0.17).

Intra-abdominal Bile Spillage

**Figure 4:**
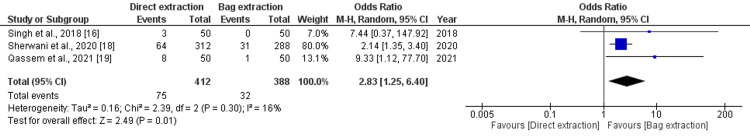
Forest plot analysis of bile spillage CI: Confidence interval

Intra-abdominal spillage of bile was reported in three studies including 800 patients. As seen in Figure 4, in comparison to the bag group, the bile spillage rate was statistically higher in the direct extraction group (18.2% versus 8.2%, OR 2.83, 95% CI (1.25,6.40), p=0.01). A low level of heterogeneity was estimated between the included studies (I2=16%, p=0.30).

Extension of the Fascial Defect

**Figure 5:**
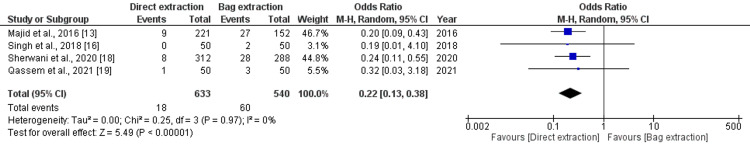
Forest plot analysis of fascial extension CI: Confidence interval

Extension of the port-site fascial defect was assessed in four studies, and the extension was required to extract the gallbladder in 78 patients (6.6%). Figure 5 shows the difference between the two groups was statistically significant in the favour of the direct extract group (2.8% in the direct extraction group versus 11.1% in the bag group, OR 0.22, 95% CI (0.13, 0.38), p=0.00001). A low level of heterogeneity was observed between the included studies (I2=0%, p=0.97).

Postoperative Intra-abdominal Collection

**Figure 6:**
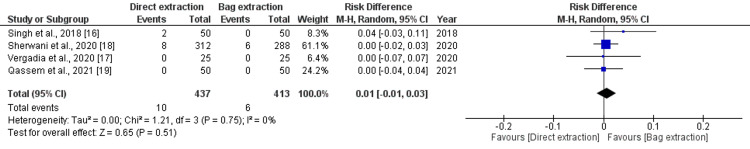
Forest plot analysis of postoperative collection CI: Confidence interval

The postoperative abdominal collection rate was reported in four studies with a total number of 850 patients, 1.88% of them (16 patients) developed postoperative abdominal collection. Figure 6 shows the difference was not statistically significant between the two groups (1.45% in the bag group versus 2.29% in the direct extraction group, OR 0.01, 95% CI (0.01- 0.03), p=0.51). The level of heterogeneity was low among the included studies (I2=0%, p=0.75).

Port-site Hernia

**Figure 7:**
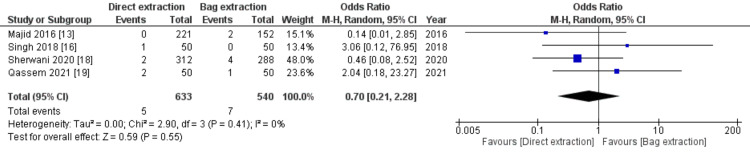
Forest plot analysis of port site hernia CI: Confidence interval

Port-site hernia was reported in four studies with a total number of 1173 patients. Twelve patients (1.02%) developed port-site hernia across the two groups, however, the comparison between the two groups showed comparable results with no significant difference (0.79% in the direct extraction group versus 1.3% in the bag group, OR 0.70, 95% CI (0.21, 2.28), p=0.55]) as seen in Figure 7. A low level of heterogeneity was calculated between the included studies (I2= 0%, p=0.41).

Sensitivity Analysis

The direction of the pooled effect size remained unchanged when RR or RD was calculated for dichotomous variables. Furthermore, the leave-one-out analysis has not demonstrated important discrepancies with the original analysis.

Discussion

Cholecystectomy is the definitive treatment for gallstone-related conditions, including biliary colic, cholecystitis, cholangitis, and biliary pancreatitis, as well as biliary dyskinesia and gallbladder cancer [7,21]. An estimated 70,000 cholecystectomy procedures are performed annually in the UK and remain one of the most common surgical operations in the US with more than 1.2 million cases per year [22,23]. Instantly, cholecystectomy has been profoundly influenced by the evolution of laparoscopy and minimally invasive surgery resulting in the widespread adoption of LC [24]. The evident benefit of laparoscopy in terms of reducing postoperative pain, shortening hospital stay, and quicker return to normal life activities, contributes to the current profile of more than 90% of cholecystectomies being done laparoscopically [25]. Despite the higher cost when using the operating theatre for laparoscopic procedures compared to the conventional open approach, the direct savings of shorter hospital stays and the indirect financial gains from the rapid return to work and activities make the laparoscopic approach deservedly sought after [26].

There are a lot of techniques and innovations that have been widely used by surgeons in response to the fast-evolving laparoscopic surgery in order to provide the best results and avoid postoperative infection risks [13,14,27]. Different types of retrieval bags have been introduced to the markets and become widely popular to provide a nice and clean surgery not just in LC but also in appendectomy, splenectomy, and adrenalectomy [15]. The popularity of retrieval bags comes from the point where some associated problems with laparoscopic surgery can be avoided such as bile spillage, port site infection, and loss of gallstones or tissue fragments following the operation. Using a bag or no bag for specimen following laparoscopic surgery is an operating surgeon’s decision, however, it is a safe and good surgical practice to use an endo-bag to avoid any risk of developing SSI despite the low level of evidence [16]. On the other hand, there are still some drawbacks that have not been addressed properly yet, such as how using endo-bags can lead to some unexpected complications at different points [17,18].

Moreover, specimen extraction in laparoscopic surgery ought to be optimized to maintain time efficiency and lessen the associated morbidity [13,17,28]. Several thoughts regarding extraction of gallbladder specimens after cholecystectomy have been explored, historically from the point of small laparotomy incision which is used to extract specimens following laparoscopic resections to a small extension of umbilical port incision of 3 cm to 5 cm to provide enough retrieval space for slightly larger specimens [19]. Meanwhile, the morcellation technique has been used to facilitate the use of smaller incisions with a few preservations in cases of cancer [20,21]. Despite the advantages of laparoscopy, the rates of SSI, wound dehiscence, and incisional hernia continued to be reported in the literature [29-32]. In further trials to lessen the potential morbidity of specimen extraction, surgeons adopted extraction through unusual ports: in the case of cholecystectomy extracting the gallbladder through the epigastric port has shown some benefits over the conventional extraction via the umbilical port due to its lower rate of port site hernia and shorter operative time [33,34]. Conversely, some studies concluded less postoperative pain with umbilical port extraction [35]. This study demonstrated a significant statistical difference between the two groups: when an endo-bag was used for gallbladder retrieval, the rate of port site fascial extension and subsequent postoperative port site hernia was notedly high. Studies by Narayanswamy et al. and Majid et al. have concluded the same results [13,21,33,36]. Hence, using the bag extraction technique is associated with difficult retrieval, longer operative time, and increased pain postoperatively in comparison to direct extraction where no fascia extension is usually required.

Surgical site infection has been reported to be around 2.4% to 3.2% following LC, where bile spillage and blood loss were deemed to be significant risk factors [36,37]. Certain factors are associated with increased frequency of gallbladder perforations, bile, and stone leakage [37]. An acutely inflamed, friable, and distended gallbladder or the presence of dense adhesions can make the gallbladder liable to tear on manipulation. Also, a slip of the cystic artery clip during operation can contribute to bile spillage and thereafter port site infection [35,36,38]. Subsequent Infectious consequences may occur early or late due to non-specific initial symptoms of wound infection or abscess formation, hence the risk of SSI and the potential challenges of extracting relatively large specimens through a small trocar site have led to the development of the retrieval bags, which have grown in popularity among surgeons of different specialties.

The use of retrieval bags not only reduces the risk of bile spillage and SSI in cases of cholecystectomy but also minimizes the seeding of cancer cells on extracting cancerous specimens [39,40]. Intraoperative gallbladder perforations, bile, and stone spillage may mandate using a retrieval bag to contain peritoneal contamination and facilitate a clean and fast retrieval process [41]. The cost of retrieval bags ranges from 25 to 120 Euros per case according to type, quality, and manufacturer; this relatively high cost can be argued especially in the current economic burden facing healthcare [42]. Few hand-made and cheaper alternatives have been introduced to seek cost-efficiency, particularly in countries with underfunded overcrowded healthcare systems [43,44].

There is still an increasing concern about specific complications of LC due to gallbladder perforation which occurs in up to 40% of cases [12,34,45]. Using an endo-bag for gallbladder retrieval has shown a statistically significant low risk of bile and stone spillage compared to a direct extraction technique [25,26,46]. Demirbas et al. have strongly recommended the endo-bag retrieval technique to minimize the risk of intra-peritoneal abscess and fistula which results from a dramatic intraoperative bile leak [26,47,48]. It was noted that postoperative intra-abdominal collection rates did not reach the statistical significance level in the current study, this can be explained by the limitation of studies included in this meta-analysis as more research is needed for further assessment.

The incidence of port-site infection in LC is unclear whether it is related to infected gallbladder or skin flora. In this study, the use of an endo-bag is directly associated with reduced incidence of port-site infection because it acts as a barrier against spillage of gallbladder contents in contrast to direct extraction where the contact between gallbladder and skin is inevitable. La Regina et al. reported a 4.2% rate of port site infection in patients operated on using a retrieval bag versus 5.9% of patients operated on with no retrieval bag, which is almost similar to what this study shows [28,35,48]. In the most of included studies, the port site infection has proven to be directly correlated to an infected gallbladder or spillage of the contents through an abdominal wall defect [13-15,17,43]. Hence, the use of retrieval bags in LC remains, in most cases, at the surgeon’s discretion [17,41,46]. While some surgeons recommended only using retrieval bags in cases of acute cholecystitis and gallbladder empyema, the increased prevalence of bile bacteria in presence of gallstones refutes this practice. Positive bile cultures range from 11% to 30% in cases of symptomatic gallstones and chronically inflamed gallbladders, and even more in elderly patients [17,42,44].

Limitations

This systematic review and meta-analysis evaluated the role of using retrieval bags when extracting gallbladder specimens in cholecystectomy surgery through pooling results from four randomized trials and four prospective studies [41,48]. The use of a retrieval bag was deemed to be superior to direct retrieval with no bag in terms of lower rates of SSI and bile spillage, whereas it was associated with a risk of fascial incision extension when extracting the specimen. There was no difference in the rest of the assessed outcomes, including abdominal collections and port site hernias.

A 2018 meta-analysis conducted in the same context could not mandate the routine use of extraction bags unless in presence of acute cholecystitis, gallbladder perforation, and suspected malignancy. A noteworthy fact in this review is that it mainly explored one outcome, wound infection, and lacked conclusions from the most recent RCTs [41]. 

Interestingly, most of the included studies (six out of eight), were executed in countries of the developing world such as India and Egypt, where cost-effectiveness is the key to supporting their overwhelmed healthcare systems [41,42,48]. The female predominance of gallstone disease is also evident in the studied population. Although our review includes up-to-date evidence, it has a few limitations. The involved RCTs excluded complicated cases such as gallbladder empyema, gangrene, and cholecystectomy in the immunocompromised populations, which compromise an unneglectable portion of our population. Meanwhile, two prospective studies included more realistic and representative populations while the other two had some exclusions [17,43]. The Egyptian comparative study excluded patients who are above 65 years old and those having American Society of Anaesthesiology (ASA) grade 3 or more, while the Irish research did not include cases of intraoperative stone or bile leak [41,43]. Additionally, there was a discrepancy in a few operative steps which may have had an impact on port-site complications. In all of the included studies, surgeons extracted the specimen through the epigastric port, apart from one study in which the umbilical port was the gallbladder retrieval port [44]. The types of extraction bags used in the studies varied from simple plastic bags and tailored surgical gloves in most of the involved studies to a more advanced retrieval device such as the Inzii retrieval system used in one study [48]. Also, port-site closure techniques and prophylactic antibiotics regimes were missing from most of the studies.

## Conclusions

This systematic review and meta-analysis evaluated the role of using retrieval bags when extracting gallbladder specimens in laparoscopic cholecystectomy surgery by pooling results from eight comparative studies. The use of a retrieval bag was deemed to be superior to direct retrieval with no bag in terms of lower rates of SSI and bile spillage, however, it was associated with a risk of fascial incision extension when extracting the specimen. There was no difference in the rest of the assessed outcomes, including abdominal collections and port-site hernias.
